# Cracking in Reinforced Concrete Cross-Sections Due to Non-Uniformly Distributed Corrosion

**DOI:** 10.3390/ma16186331

**Published:** 2023-09-21

**Authors:** Magdalena German, Jerzy Pamin

**Affiliations:** Chair for Computational Engineering, Cracow University of Technology, Warszawska 24, 31-155 Cracow, Poland; jerzy.pamin@pk.edu.pl

**Keywords:** reinforced concrete, corrosion of reinforcement, chloride concentration, finite element simulation

## Abstract

Corrosion affecting reinforced concrete (RC) structures generates safety and economical problems. This paper is focused on the simulation of corrosion-induced fractures in concrete, whereby non-uniform corrosion growth is taken into account. In particular, the volumetric expansion of rust accumulated around reinforcement bars causes cracking of the surrounding concrete. This phenomenon is simulated using the finite element (FE) method. In the analyses, concrete is described as a fracturing material by using a damage–plasticity model, steel is assumed to be elastic–plastic and rust is modeled as an interface between concrete and steel. The behavior of corrosion products is simulated as interface opening. Two-dimensional FE models of RC cross-sections with 2, 4 or 6 reinforcing bars are considered. Crack formation and propagation is examined. Moreover, interactions between cracks and patterns of possible failure are predicted. The most developed and complex crack pattern occurs around the side reinforcing bar. Conclusions concerning the comparison of results for uniform and non-uniform corrosion distribution as well as the prediction of concrete spalling are formulated.

## 1. Introduction

Chloride corrosion of reinforcement is a process that highly devastates reinforced concrete (RC) elements. The reinforced concrete structures infected by corrosion often do not meet the requirements of serviceability limit states (SLS) and sometimes also of the ultimate limit states (ULS), which in the end generates huge expenses. Volumetric expansion of rust accumulated around reinforcement bars causes cracking of the surrounding concrete. In modeling, chloride corrosion is usually represented by pressure uniformly distributed around a bar and acting on a thick-walled concrete cylinder or cubic sample [1]. However, to obtain the pattern of possible failure of an RC cross-section due to corrosion, the actual geometry needs to be modeled. It is then possible to include interactions between concrete cracks caused by more than one corroding rebar. Another issue is the rust representation. Usually, the mass of steel consumed in the corrosion process is computed with Faraday’s law [2,3,4,5]. However, the geometry of rust is rather irregular and rust is not uniformly distributed around the reinforcement. The rebars do not corrode equally fast in time and, moreover, the rust generation starts earlier in the bars placed in the corners of the cross-section than in those placed along the edges. Hence, the rebar placement needs to be included in the analysis as it results in different cracking patterns for corner rebars than the middle ones [6,7]. These problems are addressed and solved in this paper.

In the presented examples, finite element (FE) models of concrete cross-sections reinforced with 2, 4 and 6 rebars are considered. The numerical simulation of an RC element takes into account the non-uniform distribution of rust. All analyses treat concrete as non-linear, fracturing material and rust as an interface between concrete and steel. Small deformations are assumed. The expanding behavior of corrosion products is simulated as an opening of the interface between steel and concrete. The validation of the model is performed with respect to experiments presented by Andrade et al. in [1].

We start the presentation of the modeling concepts with an overview of the chloride corrosion process in RC. The pH of concrete decreases in time (due to carbonation or change in moisture content). Additionally, free chlorides present in the pore solution are responsible for breaking the passive layer on the reinforcement surface. In real-life structures, corrosion is a result of a combination of many factors. When the pH of the pore solution drops below approximately 12 and the chloride concentration is above a threshold value, the passive layer is decomposed [8] and a corrosion current starts to flow through the reinforcement. The electrical current induces the production of rust on the reinforcement surface. The rust occupies a much larger volume than the steel consumed in the process. At first, the corrosion products fill all pores and free spaces in concrete. In [9], one can learn that the porous zone (formed by voids in the concrete filled by rust in the initial stage of the process) is approximately 12.5 μm thick. In the 2D models presented in that paper, the initial thickness of rust is estimated as 20 μm; however, it is assumed that the thickness is a result of steel cross-section loss only. Afterward, the corrosion layer thickness can increase locally even to 100 μm [10,11]; hence, a significant internal pressure acting on the surrounding concrete is generated. As a result, concrete cracks outward from the rebar and the cracks propagate along the shortest path to appear on the concrete surface. Further, a longitudinal crack can occur on the whole structural element [12,13]. In the end, the corrosion process leads to crack opening, splitting, delamination and loss of strength of the element.

Although much research has been performed on the matter of corrosion, it is still difficult to determine reliably the rust properties. Its mechanical parameters, as well as the phenomena occurring at the contact surface between steel and concrete, need to be idealized. In the literature, one can find simulations with different representations of rust, using, for instance, connector elements [14], damage parameters introduced in the material model of steel and in the bond-slip relation [14,15,16] or imposed displacements at the steel–concrete contact surface [17]. In [18,19], a smeared rust layer was introduced into the model, and corrosion expansion is also simulated by relevant displacements.

Another idea is to represent the rust expansion using the thermal analogy, i.e., an equivalent increase in temperature. Such an approach enables the simulation of both uniform and non-uniform corrosion. For instance, in [6,20,21,22,23], one can find different finite element models in which temperature change is applied directly to the reinforcement to simulate non-uniformly distributed corrosion. In the present paper, a numerical model is employed [24], where rust is represented by an interface with an assumed traction–separation relation. The rust expansion is simulated by means of an increase in temperature in the cohesive interface.

The mass and density of rust can be introduced by the following relations:

(1) $$r_{m}\;M_{r}=M_{s}\;\;\;\;\;\;\;\;\;\gamma \;\rho_{r}=\rho_{s}$$

where $r_{m}$—iron-to-rust molecular weight ratio, with typical values 0.523 for $\mathrm{Fe}{\left(\mathrm{OH}\right)}_{3}$ or 0.622 for $\mathrm{Fe}{\left(\mathrm{OH}\right)}_{2}$; $M_{r}$—mass of rust in kg/m; $M_{s}$—mass of steel consumed in the process in kg/m; γ—parameter with a value usually ranging 2–4 [5]; $\rho_{r}$—rust density; $\rho_{s}$—steel density, assumed as 7890 kg/m^3^.

As mentioned before, the most common model used for the calculation of the mass of steel consumed in the process is Faraday’s law [2,3,4,5,25]. However, as the rust layer thickens, the iron diffusion rate goes down and the rate of rust production decreases [26]. In [27], Liu proposed an alternative formula, assuming a variable rate of rust production in time, also used in [5]. Balafas and Burgoyne proposed, in [28], a combined rule, assuming that initially, the corrosion rate is constant, following Faraday’s law, and later it is evaluated using Liu’s expression. The turning point is the moment when the rates of rust production calculated with both models are equal. The thorough analysis and calculations of the mass of rust have been presented in [26].

In the paper, the mass of rust is introduced into the model as so-called corrosion level $L_{corr}$, a unitless variable understood as the loss of weight related to the initial weight of a rebar, calculated according to:

(2) $$L_{corr}=\frac{r_{m}M_{r}}{\rho_{s}A_{rebar}}$$

where $A_{rebar}$ is the cross-section area of a reinforcement bar.

**Figure 1:**
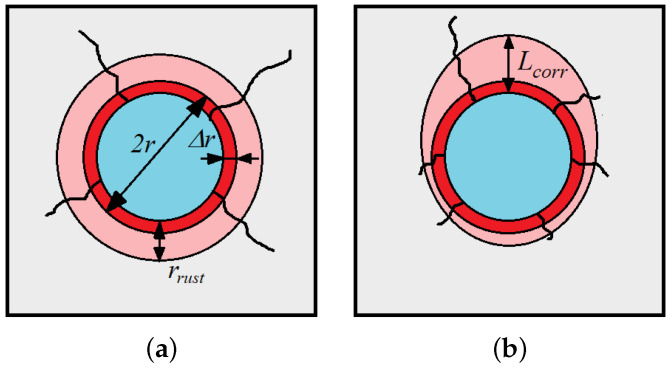
Rust volumetric expansion: (**a**) idealization of steel consumption and uniformly distributed rust, *r*—rebar radius, Δr—loss of rebar radius due to steel consumption, $r_{rust}$—rust layer thickness, (**b**) illustration of non-uniformly distributed rust, $L_{corr}$—unitless corrosion level according to Equation (2).

The location of points with passive layer decomposition is random and depends on the porosity of the concrete, properties of pore solution and mechanical influences [26]. The description of the electro-chemical process occurring in the propagation phase of corrosion can be found in [2,3,4,8,10,28,29,30]. It must be pointed out that, due to varying concrete cover carbonation and content of chloride ions around the bar, the geometry of rust is rather irregular, i.e., rust is hardly ever uniformly distributed around the reinforcement [26]. The comparison of those two situations is presented in Figure 1. It can be assumed that the corrosion starts at the first point of depassivation, in other words, at the point subjected to the highest chloride concentration, and then it propagates the way the depassivation changes [10,31]. In fact, in the case of chloride corrosion, the time to depassivation depends strongly on the threshold chloride concentration [32], but this was the subject of our previous research presented in [26].

Many analyses contribute to experimental procedures simulating electro-chemical reactions and rust generation. The laboratory tests are performed in terms of accelerated corrosion, which itself introduces some imperfections when compared to real-life situations [10,13,14,15,33]. However, due to the long-term character of corrosion processes, there are very few in situ tests and the accelerated ones can be the only solution [34,35]. An interesting connection between accelerated and long-term experiments of corrosion, incorporating additionally concrete creep, is presented in [17].

In the case of uniformly distributed corrosion, Equation (2) can be expressed in terms of reinforcement radius and its loss due to corrosion:

(3) $$L_{corr}=\frac{2r\Delta r-{\left(\Delta r\right)}^{2}}{r^{2}}$$

In the case of non-uniformly distributed corrosion, it is much more convenient to use Equation (2), as it operates on the mass of steel currently consumed in the process. This makes it possible to apply a correct level of corrosion strictly at depassivated points, without the necessity to unrealistically distribute corrosion uniformly around the reinforcement. Thus, the corrosion level is the link between the first phase of corrosion, when the environmental factors influence the intensity of rust production and the second phase when concrete cracks due to expanding rust.

The propagation of the cracks caused by corrosion has been analyzed, for instance, in [36,37,38,39,40,41,42]. A part of the research considers a single rebar corroding due to rust uniformly distributed on the reinforcement surface. To analyze the mechanical aspects of the propagation phase, Bazant [43] introduced a model representing rust expansion and its influence on surrounding concrete as a thick-walled cylinder with a thickness equal to the concrete cover. Using this model, it is not possible to predict the pattern of possible failure of the whole concrete element because the interactions caused by more than one corroding rebar are neglected. The thick-walled cylinder model has been used by other researchers [2,5,17,44,45,46]; however, in the literature, one can also find analyses of multi-reinforced cross-sections under corrosion [9,14,15,24,47]. Other techniques, like homogenization [48], artificial neural networks [49] or 3D laser scanning and digital image correlation [50], are also used nowadays for corrosion-induced cracking analyses.

This paper presents the methodology of numerical simulations of concrete cracking due to reinforcement corrosion. The computations are performed in Abaqus/CAE 2022 software. The initial simulations strongly refer to experimental data presented in [1] in order to calibrate some model parameters. The presentation of research results is organized as follows. In Section 2, the simulation framework is briefly discussed. The description considers both the computational methods and constitutive laws used for calculations. In Section 3, the simulation of a single rebar is performed to obtain the correct analysis parameters of corroding steel. Then, in Section 4, the simulation of a concrete element, reinforced with a few rebars in different configurations, is presented. The simulated crack is allowed to penetrate through the cover as well as run between the rebars. Such an approach is a much more realistic representation of the damaging effect of rust then the consideration of samples with a single rebar. Furthermore, the analysis of an RC element with non-uniformly distributed corrosion is presented in Section 5. Finally, Section 6 provides some discussion and conclusions.

## 2. Simulation Framework

### 2.1. Rust Interface Model

**Figure 2:**
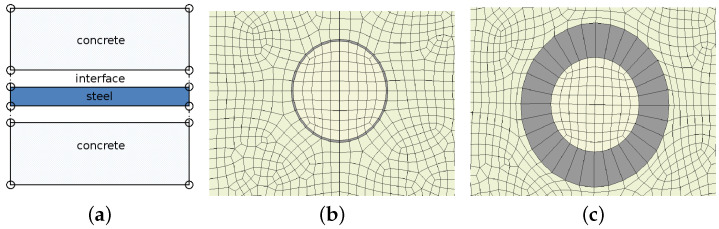
Continuum model of steel and concrete, and corrosion introduced as interface elements: (**a**) general idea, (**b**) simulation with closed interface (no corrosion), (**c**) simulation with open interface (growing corrosion).

In the paper, the expanding corrosion products are represented by interface elements placed between respective solid materials. The corrosion interface is shown in Figure 2.

**Figure 3:**
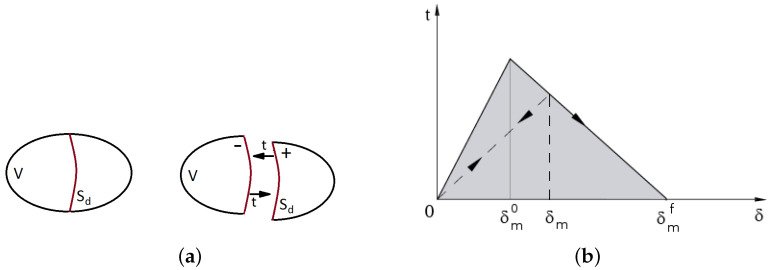
Interface: (**a**) example representation with normal traction t, (**b**) linear softening in traction–separation description [51].

The volumetric expansion of rust is introduced into the simulation as an opening interface. It represents a discontinuity in the FE model, as presented in Figure 3a. The response of the cohesive interface is defined in terms of traction versus separation and described in [51]. This model initially assumes the linear elastic behavior followed by initiation and evolution of degradation [52,53]. The elastic behavior is described in terms of an elasticity matrix that relates the nominal tractions to the nominal separations across the interface. The nominal traction vector consists of three components: one normal component $t_{n}$ and two shear components $t_{s}$, $t_{t}$. The corresponding separations are denoted by $\delta_{n}$, $\delta_{s}$ and $\delta_{t}$, respectively. If no couplings are assumed, the elastic relations can be written as:

(4) $$\left(\begin{array}{c} t_{n}\\ t_{s}\\ t_{t} \end{array}\right)=\left[\begin{array}{ccc} K_{nn} & 0 & 0\\ 0 & K_{ss} & 0\\ 0 & 0 & K_{tt} \end{array}\right]\;\left(\begin{array}{c} \delta_{n}\\ \delta_{s}\\ \delta_{t} \end{array}\right)$$

In the calculations, the initial thickness $h_{0}$ of rust is assumed to be 0.02 mm. The initial non-zero thickness of the interface is very useful in mesh generation, and also affects the stiffness matrix in Equation (4). The traction separation is linear until reaching the damage initiation criterion. In the presented model, the maximum nominal stress criterion is used, cf. [51]:

(5) $$\max\left\{\frac{\langle t_{n}\rangle }{t_{n}^{0}},\frac{t_{s}}{t_{s}^{0}},\frac{t_{t}}{t_{t}^{0}}\right\}=1$$

where $t_{n}^{0},t_{s}^{0},t_{t}^{0}$—respective limit values of the nominal traction components when the deformation is purely normal or in one of the shear directions. Damage to the interface is assumed to initiate when the maximum nominal traction ratio reaches 1, as defined in Equation (5). The expression with MacAuley brackets $\langle t_{n}\rangle$ means that damage occurs only when the normal traction is tensile.

After reaching the peak value, a linear softening behavior in the traction–separation relation is considered. It is introduced by variable *D*, which represents the overall damage in corrosion products and captures the combined effects of all active mechanisms. The damage of the interface was investigated in [54]. The interface itself does not represent the rust as a material. It describes the concrete-steel relationship infected by corrosion. Hence, the damage in the simulation is not the physical deterioration of the material. It is the reduction of the traction–separation relation on the concrete-corroded steel contact surface, which is especially important when the bond-slip relation is analyzed [54].

The traction components are affected by damage according to the following relations:

(6) $$\begin{array}{c} t_{n}=\left\{\begin{array}{c} \left(1-D\right)\cdot {\hat{t}}_{n}\;\;\;\;\;\;\mathrm{for}\;\mathrm{tension}\\ {\hat{t}}_{n}\;\;\;\;\;\;\mathrm{for}\;\mathrm{compression} \end{array}\right.\\ t_{s}=\left(1-D\right)\cdot {\hat{t}}_{s}\\ t_{t}=\left(1-D\right)\cdot {\hat{t}}_{t} \end{array}$$

where: ${\hat{t}}_{n}$, ${\hat{t}}_{s}$, ${\hat{t}}_{t}$—effective traction components predicted by the elastic relation for the current strains (without damage).

The effective separation is defined as:

(7) $$\delta_{m}=\sqrt{{\langle \delta_{n}\rangle }^{2}+\delta_{s}^{2}+\delta_{t}^{2}}$$

The damage evolution is described in terms of effective separation limits:

(8) $$D=\frac{\delta_{m}^{f}\left(\delta_{m}-\delta_{m}^{0}\right)}{\delta_{m}\left(\delta_{m}^{f}-\delta_{m}^{0}\right)}$$

where: $\delta_{m}^{f}$—effective separation at complete failure, $\delta_{m}^{0}$—effective separation at damage initiation and $\delta_{m}$—effective separation attained during the loading history. The softening behavior adopted from [51] is presented in Figure 3b.

The previous research [54,55] showed that interface damage is very important when the shear components at high levels of corrosion are activated. When the response in the normal direction dominates the interface, the overall failure of an RC element is a result of cracking of concrete and the normal damage in the interface can be neglected. In the physical process, the two key parameters limiting rust production are oxygen and iron supplies. Thus, in a favorable environment with enough oxygen, rust can be produced until the iron source becomes exhausted. Since rust is produced constantly and it is assumed that there is no correlation between the normal and shear tractions, it is hard to define the limit of normal traction $t_{n}$. Thus, the value $t_{n}^{0}$ can be assumed to be an arbitrarily high number.

### 2.2. Concrete and Steel Continuum Models

The constitutive model for concrete used in the calculations is based on the plasticity-damage formulation presented in [56,57], implemented in Abaqus, called the concrete damage-plasticity (CDP) model and briefly reviewed below. The model assumes that the main two failure mechanisms are cracking and crushing; hence, the material characteristics are defined separately for tension and compression. The material degradation associated leads to a reduction of the initial elastic stiffness. Scalar damage variables for tension and compression $\left\{d_{t},d_{c}\right\}$, with the values changing within the range $0\leq d_{t/c}\leq 1$, are introduced to relate the reduced secant stiffness operator and the elastic stiffness matrix $D^{e}$. This allows one to express the stress in tension/compression $\sigma_{t/c}$ in terms of effective stress ${\hat{\sigma }}_{t/c}$ acting on the undamaged skeleton of the material:

(9) $$\sigma_{t/c}=\left(1-d_{t/c}\right){\hat{\sigma }}_{t/c}=\left(1-d_{t/c}\right)D^{e}\left(\varepsilon_{t/c}-\varepsilon_{t/c}^{p}\right)$$

where: $\varepsilon_{t/c}$—total strain in tension/compression, $\varepsilon_{t/c}^{p}$—plastic part of strain in tension/compression.

In the CDP model available in Abaqus, the yield function *F* is a function of effective stress $\hat{\sigma }$ and hardening variables ${\tilde{\varepsilon }}^{p}$:

(10) $$F(\hat{\sigma },{\tilde{\varepsilon }}^{p})\leq 0,$$

**Figure 4:**
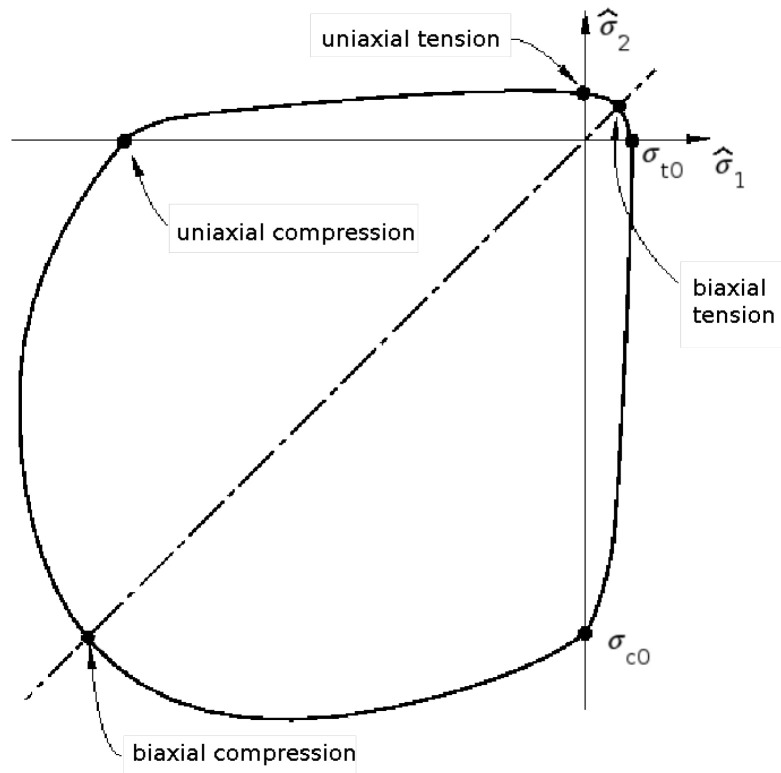
Initial yield surface of concrete damage–plasticity model in principal effective stress space for plane stress [51].

The following Kuhn–Tucker conditions are satisfied:

(11) $$\dot{\lambda }F=0\;,\;\;\;\;\;\;\;\;\;\;\dot{\lambda }\geq 0\;,\;\;\;\;\;\;\;\;\;\;F\leq 0$$

where: $\dot{\lambda }$—non-negative plastic multiplier. The initial yield surface for plane stress conditions is presented in Figure 4, replicated from [51]. Under uniaxial tension, after reaching the initial tensile limit stress, $\sigma_{t0}$, the formation of micro-cracks is represented macroscopically with a softening stress–strain response. Under uniaxial compression, the response is linear until the value of initial compressive strength is reached, $\sigma_{c0}$. In the plastic regime, the response is typically characterized by yield stress hardening, followed by strain softening beyond the ultimate stress [51].

The plastic flow is governed by flow potential Φ, defined in the effective stress space, and the non-associated flow rule:

(12) $$\Phi =\sqrt{{\left(\epsilon \;\sigma_{t0}\;\tan\left(\psi \right)\right)}^{2}+{\hat{q}}^{2}}-\hat{p}\;\tan\left(\psi \right)\;,\;\;\;\;\;\;\;\;\;\;{\dot{\varepsilon }}^{p}=\dot{\lambda }\frac{\partial \Phi (\hat{\sigma })}{\partial \hat{\sigma }}$$

where: $\hat{p}$—effective hydrostatic pressure, $\hat{q}$—Mises equivalent effective stress, ϵ—parameter referred to as the eccentricity, ψ—dilation angle. The importance of adopting proper values of model parameters, in particular of the dilation angle, is discussed in [58].

The evolution of the degradation variable $d(\hat{\sigma },{\tilde{\varepsilon }}^{p})$ is governed by a set of hardening variables ${\tilde{\varepsilon }}_{t}^{p}$ and ${\tilde{\varepsilon }}_{c}^{p}$, which are referred to as equivalent plastic strains in tension (*t*) and compression (*c*), and the effective stress. Since the responses related to tensile and compressive damage are different in concrete, the model implemented in Abaqus characterizes the damage states independently in tension and compression:

(13) $$d_{t}=d_{t}\left({\tilde{\varepsilon }}_{t}^{p}\right),\;\;\;\;\;\;\;\;\;\;d_{c}=d_{c}\left({\tilde{\varepsilon }}_{c}^{p}\right)$$

The model is equipped with viscoplastic regularization according to a generalization of the Devaut–Lions approach, see e.g., [53], in which a viscous upgrade of the plastic strain tensor and hardening variables is performed using a viscosity parameter, called relaxation time μ, cf. [51]. This provides an additional ductility for the model and is an efficient method to overcome the problems with convergence of the cracking simulation algorithm.

Steel is modeled with the classical elastic–plastic model [51]. Elasticity is isotropic and linear. The Huber–Mises yield function, associated flow rule and isotropic hardening are assumed in the rate-independent plasticity description.

### 2.3. Numerical Model

All simulations are performed using the finite element method (FEM). Two-dimensional models of concrete cross-section with steel reinforcement are built in Abaqus/CAE 2022 software within its standard version. In general, the calculation models are composed of three parts, concrete, rust and steel, in different configurations, as presented in Figure 2.

The Abaqus calculations are performed for meshes with the approximate size of an element 0.5 mm. The mesh is composed of 4-node plane strain elements for the concrete and steel parts. The rust interface is modeled with 4-node cohesive elements. Since the component materials are represented as inelastic, the Newton–Raphson algorithm is used in the nonlinear computations with an implicit time integration scheme.

Another problem to be solved is to create a credible way of applying rust expansion. All three parts are tied together so that all degrees of freedom are transferred from one part to another. The corrosion-induced internal pressure is applied to the surrounding concrete by using substitute thermal expansion of rust. Thus, an artificial temperature increase interpreted as corrosion level is applied to the rust interface. The whole behavior is then governed by expansion parameter α:

(14) $$\frac{\delta_{n}}{h_{0}}=\alpha \;\Delta T$$

where: ΔT is the substitute increase in the temperature in the rust layer.

## 3. Initial Simulations

**Figure 5:**
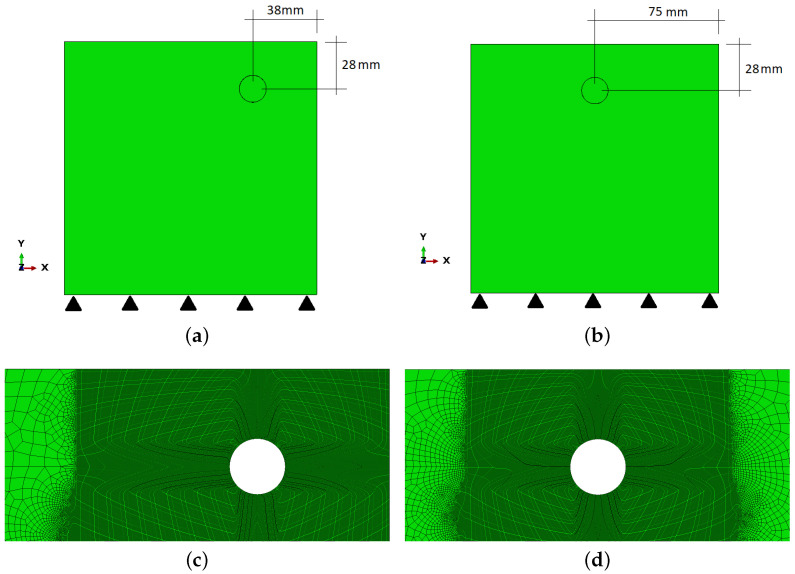
Configurations of models Sp1 (**a**) and Sp2 (**b**), mesh composition of their respective upper parts, (**c**,**d**), respectively.

To establish the proper value of α, a model calibration has been performed in accordance with the results presented in [1]. Those experimental results proved that the loss of reinforcement radius of 20 μm corresponds to the crack width (on the concrete surface) of about 0.1 mm. Simulations of the two specimens described in [1] have been performed, and the configurations of those models can be seen in Figure 5.

The analyzed specimens Sp1 and Sp2 have a square shape and dimensions 150 × 150 mm. The displacement boundary conditions are applied at the bottom edge so as not to affect the computation results. The reinforcement radius is 8 mm, but the assumed initial rust layer thickness is 0.02 mm, so that the effective radius is 7.98 mm. The concrete covers are 20 mm and 30 mm for the top and side edges, respectively, in case Sp1, while in case Sp2, the cover is 20 mm and the reinforcement is placed in the middle of the specimen width. The configurations and meshes used in the computations of cases Sp1 and Sp2 are presented in Figure 5, while material data used are listed in Table 1 and Table 2. Some of the parameters (E, ν, compressive and tensile strengths) in Table 1 are taken from [1], as the analysis refers to the experiments presented in that paper. The other parameters are taken from our previous research. An important parameter is viscosity μ. In fact, the CDP model has problems with convergence during cracking simulations; hence, it is necessary to use non-zero μ to stabilize the computations. On the other hand, its value cannot be too high since it would affect the results significantly. The value of the viscosity parameter presented in Table 1 is the smallest that allows one to prevent convergence problems in the numerical experiments presented in this paper.

**Figure 6:**
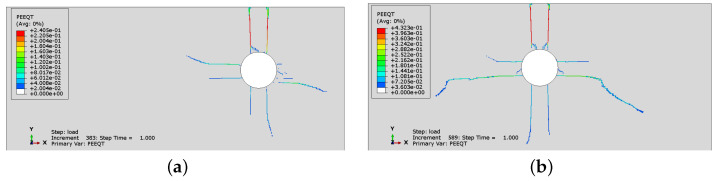
Tensile equivalent plastic strain (PEEQT) distributions without damage: (**a**) Sp1; (**b**) Sp2.

For the purpose of simulation, the loss of radius is expressed as the corrosion level according to Equation (3). This means that the 0.20 μm loss of rebar radius generates the corrosion level of 0.5%. Such a level is applied to Sp1 and Sp2, and the computations are repeated with different α values until the crack width on the concrete surface is approximately 0.1 mm. The crack width or, in other words, the crack mouth opening displacement (CMOD) is calculated as a difference between horizontal displacements of respective nodes on two sides of a crack in elements on the concrete surface, at which the tensile equivalent plastic strain (PEEQT) is monitored. For cases Sp1 and Sp2, CMOD is monitored for two cracks (left and right) because it is hard to definitely determine which one is the first to reach the concrete surface, although the left one seems to propagate quicker (see Figure 6a). In the analysis, PEEQT is monitored, since it is a suitable indicator of cracking.

In Figure 6a, it can be seen that in specimen Sp1 there are concrete cracks along the shortest path from the rebar to the surface. Two vertical cracks are observed. The horizontal crack does not reach the element surface; however, 2/3 of the cover is cracked. In Figure 6b, when the reinforcement is placed in the middle of the sample width (Sp2), the cracking pattern is almost symmetrical. The viscous regularization is on and the value of the viscosity parameter is large enough to provide stable calculations, but small enough not to disturb their final results. Again, the left vertical crack reaches the concrete surface quicker (Figure 6a); however, this is just the effect of mesh composition, without a physical reason.

**Figure 7:**
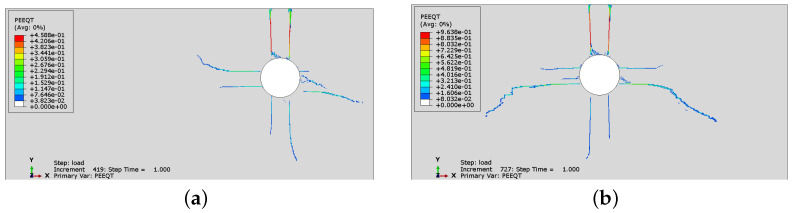
Tensile equivalent plastic strain (PEEQT) distributions with damage incorporated: (**a**) Sp1; (**b**) Sp2.

The simulation has been performed using the concrete model considering only plasticity or the plasticity–damage description. In the second variant, the values of damage parameters are assumed within a range $0\leq d_{t/c}\leq 0.5$. In Figure 6 and Figure 7a, a comparison of PEEQT distributions calculated using the plasticity or plasticity–damage model is presented. The cracking pattern, represented by the PEEQT distribution, is nearly the same. The difference is visible in the values of the plastic strain measure. In the case of the plasticity–damage model, the values were higher. According to the material description (Section 2.1), the cracking pattern depends on the plasticity part, while the damage part provides an additional reduction of stiffness in the material model; hence, higher values of PEEQT are observed in Figure 7.

**Figure 8:**
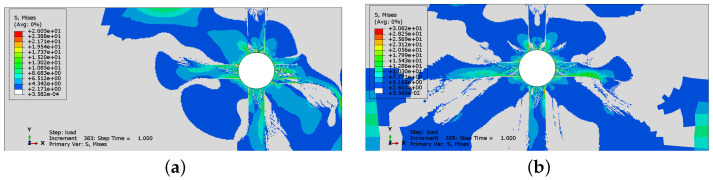
Mises stress distributions for analysis without damage: (**a**) Sp1; (**b**) Sp2.

**Figure 9:**
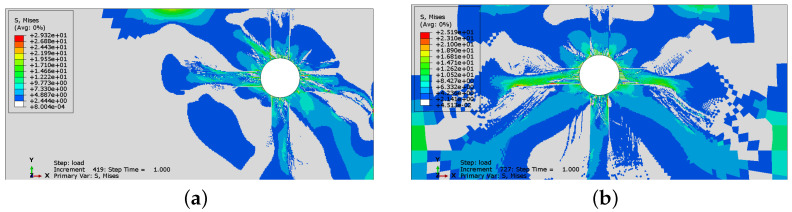
Mises stress distributions for analysis with damage incorporated: (**a**) Sp1; (**b**) Sp2.

In Figure 8 and Figure 9, the comparison of Mises equivalent stresses calculated using two options of the concrete model is presented. A stress relaxation in cracked areas can be observed in both cases. The state of stress in the results for the plasticity–damage model is more localized in specimen Sp1. On the other hand, in specimen Sp2, the non-zero stresses occupy a larger area when damage is activated. Again, the main difference is visible in the values and not in the distribution of stresses. The incorporation of the damaged part of the model decreases the values of stresses.

Since the damaged part does not affect the cracking pattern and the assumed corrosion generates monotonically increasing loading, the rest of the simulation assumes only the plasticity part of the constitutive model, i.e., the damage mode is not activated.

**Figure 10:**
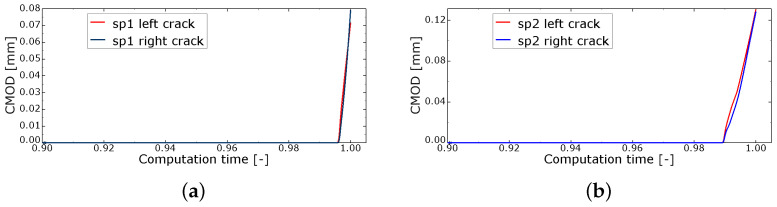
Crack mouth opening displacement (CMOD) obtained in analysis without damage counterpart: (**a**) Sp1; (**b**) Sp2.

Finally, it turns out that α = 91 for Sp1 (later referred to as side rebar) and α = 90.5 for Sp2 (middle rebar) give satisfactory results when the crack width is concerned, as presented in Figure 10. Those values of α are used in further computations distinguishing between side and middle placement of the rebar. The application of substitute temperature increase should be interpreted as a numerical procedure, which allows one to model the increase in the rust layer volume, and can be related to material parameters, such as bond strength, bond stiffness or post-failure softening.

**Figure 11:**
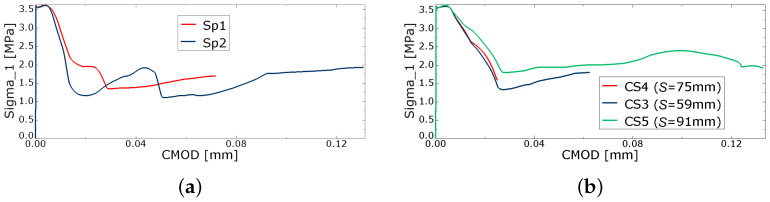
Relation of stress $\sigma_{1}$ vs. CMOD (**a**) Sp1, Sp2; (**b**) CS3, CS4, CS5.

Figure 11 presents the relation of maximum principal stress $\sigma_{1}$ vs. CMOD, monitored at the node on the concrete surface. Since, in both specimens Sp1 and Sp2, the left crack tends to be the first visible on the surface, the corresponding CMOD values are used in Figure 11a. Figure 11b presents the relation of stress $\sigma_{1}$ vs. CMOD for the specific configurations of full cross-section analysis presented in the next section.

## 4. Cross-Section Analysis—Multi-Rebar Simulation

**Figure 12:**
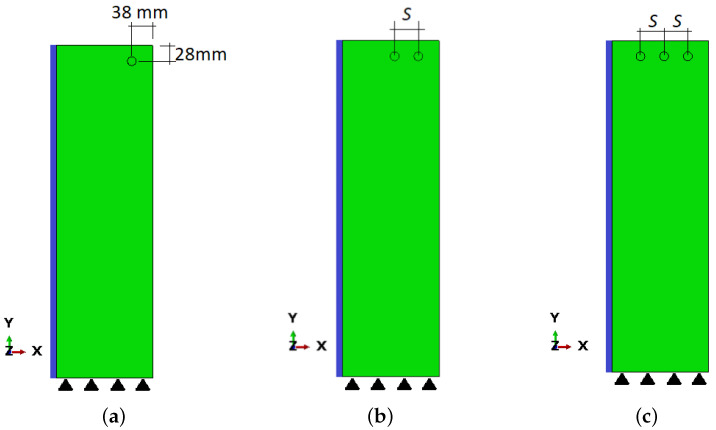
Configurations of cross-section reinforced with a growing number of rebars with different spacing: (**a**) CS1; (**b**) CS2–CS5; (**c**) CS6.

The material data employed for specimens Sp1 and Sp2 are also used in the simulation of realistic rectangular concrete cross-sections with different reinforcement positions according to Figure 12. The full cross-section dimensions are 350 × 600 mm, but for the sake of simplicity, the right half of it is analyzed. The symmetry line with the appropriate boundary conditions is in the left edge of the model, marked with the blue line, while both displacement vector components are restrained at the bottom edge, marked with black triangles, as presented in Figure 12.

In Figure 12a, the reinforcement is placed near the corner with the same cover values as in case Sp1 (20 mm and 30 mm from the top and right edges, respectively). In Figure 12b, the beam is reinforced with four rebars, of which only two are analyzed due to symmetry; however, the reinforcement spacing *S* varied in computations. The particular values of *S* can be found in Table 3. Finally, in Figure 12c, a cross-section reinforced with six bars (three analyzed due to symmetry) is presented. The radius of reinforcement is 8 mm, but due to the rust layer, the effective radius is 7.98 mm.

**Figure 13:**
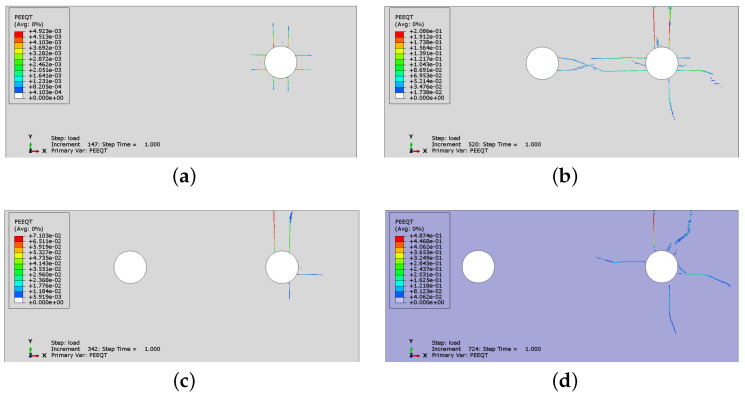
Final distributions of PEEQT for four cases: (**a**) CS1, (**b**) CS3, (**c**) CS4, (**d**) CS5 (results for cases CS2 and CS6 are presented in Figure 14 and Figure 15).

Again, the tensile equivalent plastic strain (PEEQT) distributions indicate the zones where cracking of concrete occurs. Figure 13a presents the final distribution of PEEQT for case CS1. Although the cover, reinforcement dimensions and corrosion level are the same as in case Sp1, and the cracking is less developed than the one presented in Figure 6a. There are two vertical cracks propagating through the concrete cover; however, none of the cracks are visible on the concrete surface.

Figure 13b presents the final distribution of PEEQT for case CS3 when reinforcement spacing is 59 mm. There can be observed a fully developed horizontal crack and a vertical crack visible on the concrete surface. There is no vertical crack in the case of middle reinforcement, which means there is no superposition of results of cases Sp1 and Sp2.

Figure 13c,d present the final distribution of PEEQT for cases CS4 and CS5, when the reinforcement spacing is either 75 mm or 91 mm. Again, no superposition of results of cases Sp1 and Sp2 occurs. What is more, no cracks are observed around the middle reinforcement. In case CS4, two vertical cracks are visible on the concrete surface, while in case CS5, the corner tends to spall off. The concrete around the middle reinforcement seems to be untouched by corrosion.

**Figure 14:**
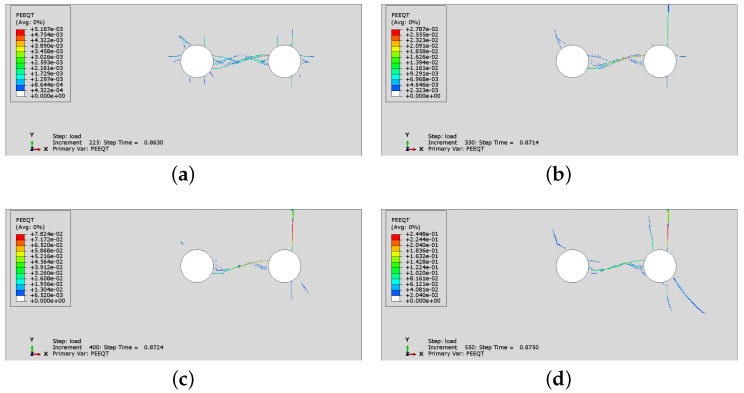
History of crack development (evolution of PEEQT) for case CS2: (**a**) state 1, (**b**) state 2, (**c**) state 3, (**d**) state 4.

**Figure 15:**
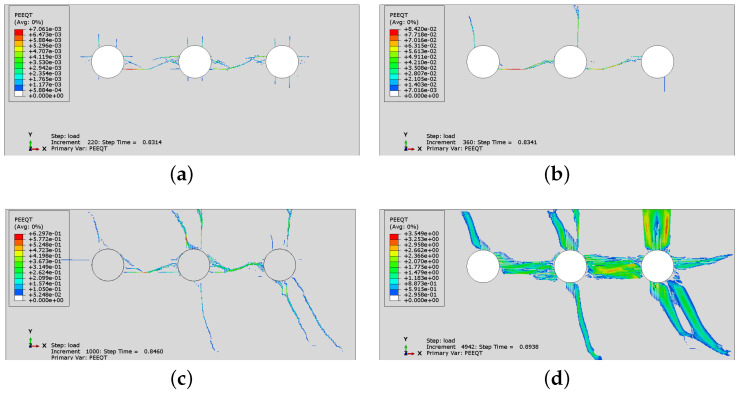
History of crack development (evolution of PEEQT) for case CS6: (**a**) state 1, (**b**) state 2, (**c**) state 3, (**d**) state 4.

In Figure 14 and Figure 15, the crack development for cases CS2 and CS6 is presented by monitoring the distribution of PEEQT for four characteristic states in the history of cracking. In both cases, the spacing is 43 mm, but the number of rebars is different. The first crack is formed horizontally between the reinforcement bars. Later, the vertical crack visible on the concrete surface appears. In case CS6, the vertical crack does not appear at first near the side rebar, but propagates from the second rebar through the concrete cover. Nevertheless, the significant horizontal crack penetrates between all rebars, which may lead to complete spalling off of the whole concrete cover, impairing the concrete confinement of the reinforcement.

## 5. Non-Uniform Corrosion Distribution

**Figure 16:**
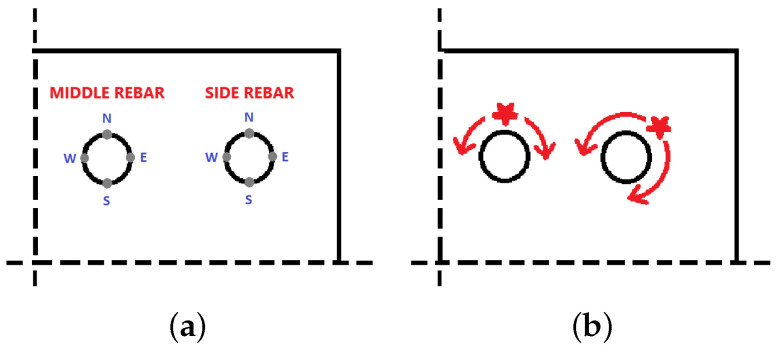
Points of application of non-uniform corrosion: (**a**) orientation of points from Table 4, (**b**) directions of corrosion propagation.

The same configurations (CS1–CS6) and FE meshes are used again, but the corrosion is non-uniformly distributed around the reinforcement, as well as non-linearly applied in time. The non-uniform distribution of rust expansion is realized by the non-uniform distribution of temperature. The corrosion levels are applied in subsequent steps of the analysis at specific points on reinforcement circumference and presented in Table 4. The points of loading application are presented in Figure 16. The data presented in Table 4 have been calculated according to the model presented in [26].

**Figure 17:**
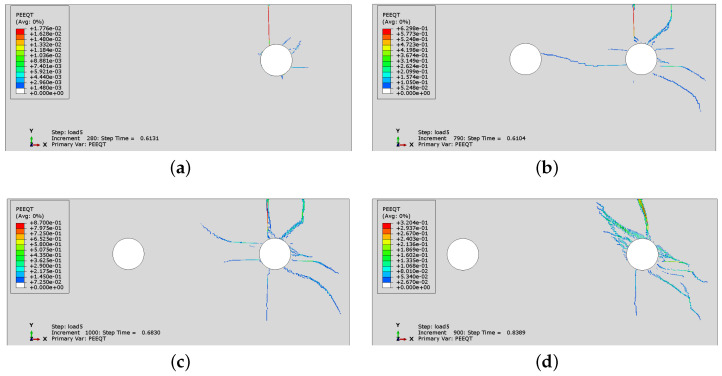
Comparison of final PEEQT distributions for cases (**a**) CS1, (**b**) CS3, (**c**) CS4 and (**d**) CS5, with non-uniform corrosion assumed.

The cracking patterns obtained for the cases CS1, CS3, CS4 and CS5 with non-uniform corrosion are presented in Figure 17. The PEEQT distribution for case CS1 (Figure 17a) is different than the one obtained for uniform corrosion (Figure 13a) and for Sp1 (Figure 6a). For non-uniform corrosion, there is one vertical crack visible on the concrete surface, which cannot be observed for uniform corrosion.

The cracking patterns for cases CS3–CS5, presented in Figure 17, depend on reinforcement spacing, similarly to the case of uniform corrosion. However, in the case of non-uniform corrosion, despite the fact that the meshes are the same, the cracking is more advanced and cracks are visible on the concrete surface. For CS4, when the reinforcement spacing is 75 mm (Figure 17c), concrete is partly cracked horizontally between the rebars, which is not observed in Figure 10c.

**Figure 18:**
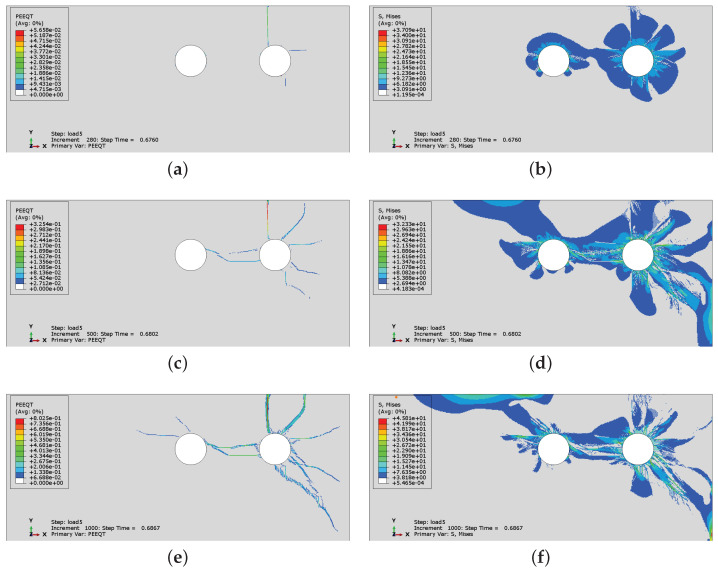
History of crack development: (**a**) state 1, (**c**) state 2, (**e**) state 3, and respective Mises equivalent stress distributions: (**b**) state 1, (**d**) state 2, (**f**) state 3 for case CS2, when non-uniform corrosion is applied.

In Figure 18, the history of crack development and respective Mises equivalent stresses are presented for case CS2 when non-uniform corrosion is applied. In case CS2, with non-uniform corrosion, a change in the cracking process occurs. The vertical crack is the first to be generated, see Figure 18a. Later, as the corrosion level increases, a horizontal crack between the rebars appears. This is different than the results presented in Figure 14, where the horizontal crack was the first to be observed, and later followed by the vertical one. In Figure 18b,d,f, the stress distribution can be observed. One can see that the areas in which stress relaxation takes place correspond to the areas in which cracking occurs.

**Figure 19:**
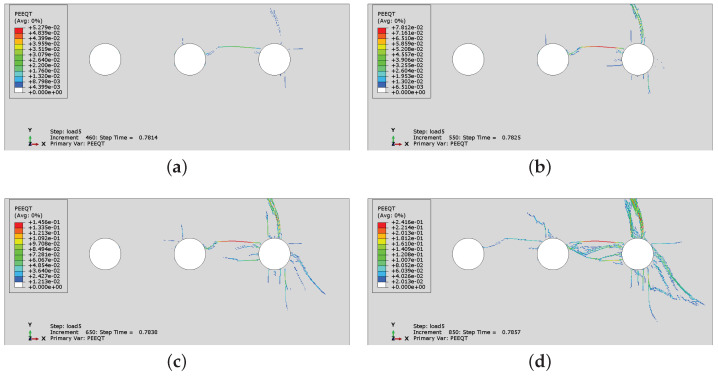
History of crack development for case CS6 with non-uniform corrosion applied: (**a**) state 1, (**b**) state 2, (**c**) state 3, (**d**) state 4.

Figure 19 presents the history of crack development for case CS6 when non-uniform corrosion is applied. As can be seen in Figure 19a, it seems that concrete cracks through the cover at the beginning of the process, although a horizontal crack between two rebars is also created. Next, Figure 19b–d shows that a vertical crack is clearly visible on the concrete surface and the horizontal crack propagates between the rebars; however, the horizontal cracking is less advanced than in the case of uniform corrosion. The non-uniform corrosion can result in spalling off of the corner of the RC element (Figure 19d), but not necessarily of the whole cover. In Figure 15c, when uniform corrosion is analyzed, the horizontal crack connects all rebars, which reduces the bond between concrete and reinforcement.

## 6. Conclusions

In this paper, the issue of concrete cracking due to reinforcement corrosion has been analyzed. The simulations have been performed in Abaqus, and concrete has been modeled using the damage–plasticity material description (so-called CDP model), with steel as the elastic–plastic material and rust as a cohesive interface. The expanding character of rust has been simulated using substitute thermal expansion of the interface with a traction–separation law. The plastic strains in concrete represent the cracks induced by the expanding interface. The concrete damage–plasticity model needs to be regularized with a viscosity parameter; otherwise, the calculations diverge prematurely.

The presented simulations refer to experimental data presented in [1]. The numerical analysis has been performed for two configurations, Sp1 and Sp2, reinforced with a single steel bar, and later for configurations CS1–CS6 of a more realistic RC element cross-section reinforced with 2, 4 or 6 bars with different spacing (and appropriate symmetry conditions). The corrosion has been applied as uniformly distributed and non-uniformly distributed around the reinforcement. The simulation of the multi-reinforced cross-section with non-uniformly distributed corrosion is the model closest to a real-life situation. The most developed and multi-directional cracking occurs around the side rebar.

In addition to concrete mechanical parameters, the cracking pattern depends also on the spacing of the reinforcement bars. As the spacing decreases, the horizontal crack between rebars is more likely to occur. This can cause spalling off of the whole concrete cover, and then the bond between steel and concrete is impaired. The horizontal crack is observed both for uniform and non-uniform corrosion; however, in non-uniform corrosion simulations, the horizontal crack is less developed. In this case, the cracks are concentrated mainly around the side rebar, causing the corner of the cross-section to spall off.

It is worth noticing that for configurations CS4 and CS5, when the bar spacing is 75 or 91 mm, hardly any cracking has been observed around the middle rebar. Thus, for a sample reinforced with many bars, the cracking pattern is not a simple combination of cracks predicted in single rebar specimens, because the processes occurring in the vicinity of the two bars influence each other. It is also mentioned that, in spite of stress redistribution taking place in the cracking history, the influence of the damage component of the CDP model is negligible in the presented simulations.

It is emphasized in this context that the proposed modeling methodology enables numerical experiments to support the optimization of the reinforcement bar placement in the beam cross-section. The future work plans contain an extension of the cross-section models to a three-dimensional representation of a reinforced concrete beam. Moreover, the influence of corrosion on the steel–concrete bond-slip relation needs to be investigated.

## Figures and Tables

**Table 1:** Material parameters.

Concrete		Steel	
Parameter	Value	Parameter	Value
E [GPa]	36	E [GPa]	210
ν [-]	0.2	ν [-]	0.3
Dilation angle ψ [°]	25	Yield stress $f_{y}$ [MPa]	350
Eccentricity ϵ	0.1		
Comp. yield stress $\sigma_{c0}$ [MPa]	40		
Inelastic strain	0.015		
Tens. yield stress $\sigma_{t0}$ [MPa]	3.55		
Cracking strain	0.02		
Viscosity parameter μ [s]	10 × 10^−6^		

**Table 2:** Rust interface parameters.

Parameter	$K_{\mathrm{nn}}$	$K_{\mathrm{ss}}$	$K_{\mathrm{tt}}$	α	$\delta_{n}$	$\delta_{s}$	$\delta_{t}$
Value [MPa]	120	50	50	91	355	17	17

**Table 3:** Details of reinforcement placement in particular configurations.

CS1	CS2; *S* = 43 mm	CS3; *S* = 59 mm
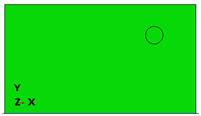	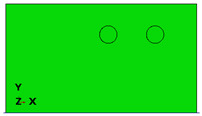	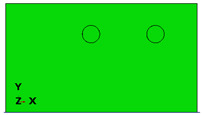
CS4; *S* = 75 mm	CS5; *S* = 91 ;mm	CS6; *S* = 43 mm
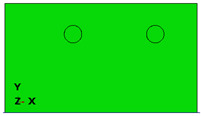	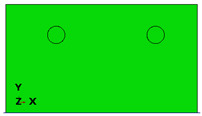	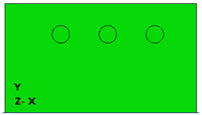

**Table 4:** Non-uniformly distributed values of corrosion level $L_{corr}$ [%].

Point	Step 1	Step 2	Step 3	Step 4	Step 5
NE (side)	0.0476	0.2252	0.3328	0.4068	0.6146
N, E (side)	-	0.1789	0.2969	0.3757	0.5919
NW, SE (side)	-	0.1576	0.2803	0.3616	0.5817
W, S (side)	-	-	0.1353	0.2422	0.5018
SW (side)	-	-	-	0.1115	0.4249
N, NW, NE (middle)	-	-	1353	0.2422	0.5018
W, E (middle)	-	-	-	-	0.3533

## Data Availability

Additional information about the input data and simulation results is available from the first author upon e-mail request.

## Abbreviations

The following abbreviations are used in this manuscript:

RC	Reinforced concrete
FE	Finite element
CDP	Concrete damage–plasticity
PEEQT	Tensile equivalent plastic strain
CMOD	Crack mouth opening displacement
